# COVID-19 Distribution in Pregnancy, Drug Use Patterns and COVID-19 Medication during the Pandemic in Spain: Data from Real-World Electronic Health Records

**DOI:** 10.3390/ph17020207

**Published:** 2024-02-06

**Authors:** Mercedes Mota-Pérez, Consuelo Huerta-Álvarez, Ana Llorente, Lucía Cea-Soriano

**Affiliations:** 1 Department of Public Health and Maternal Child Health, Faculty of Medicine, Complutense University of Madrid, Plaza Ramón y Cajal, s/n. Ciudad Universitaria, 28040 Madrid, Spain; mammota@ucm.es (M.M.-P.); tlcea@ucm.es (L.C.-S.); 2 Base de datos para la Investigación Farmacoepidemiológica en el Ámbito Público (BIFAP), Division of Pharmacoepidemiology and Pharmacovigilance, Spanish Agency for Medicines and Medical Devices (AEMPS), 28022 Madrid, Spain; allorente_externo@aemps.es

**Keywords:** drug, medication, pregnancy, pregnant women, COVID-19, Spain

## Abstract

Although pregnant women were considered a risk population for COVID-19, little is known of their drug use during the pandemic. We aimed to investigate COVID-19 distribution, drug use patterns and COVID-19 medication. We conducted a retrospective cohort of validated pregnancies aged 15–49 years, from January 2020 to December 2022, using the BIFAP database. An identified cohort of pregnant women with COVID-19 was matched by age, gestational age, length of pregnancy and outcome to a cohort free of COVID-19 (8413 vs. 24,975). We performed a descriptive analysis on COVID-19 cases, estimated the drug use patterns and assessed COVID-19-specific drugs within the week prior/after diagnosis, stratified by pandemic wave and gestational week. The results showed that 72% of pregnant women with COVID-19 received at least one prescription vs. 66.6% of those free of COVID-19, with analgesics, antibiotics and thyroid hormones being the most prescribed drugs in both groups. In the COVID-19 group, they were antithrombotics (40 prescriptions per 100 women), analgesic/NSAIDs (19.64/6.29) and antibiotics (6.95). COVID-19 cases gradually increased, peaking at the fifth and second waves. Prescription rates were similar when compared to pre-pandemic studies. The use of drugs compatible with COVID-19 treatments was in line with recommendations.

## 1. Introduction

Since the beginning of the COVID-19 pandemic, pregnant women have been a target of a risk factor population. Growing evidence has concluded that pregnant women with COVID-19 had a higher risk of maternal outcomes, such as developing respiratory complications and requiring intensive care, and perinatal outcomes including preterm birth, preeclampsia, cesarean, perinatal death and other adverse pregnancy outcomes [1,2,3,4,5]. In addition to this increased risk, during the COVID-19 pandemic, pregnant women and their offspring might have experienced more difficulties in accessing clinical services, including monitoring, antenatal visits and pharmacological treatment, among others, due to several factors such as lockdown, travel restrictions and fear of infection. Prior studies have found an inadequate utilization of healthcare services and a delay in the initiation of antenatal care visits [6,7,8]. This fact might be of special relevance as the potential cause of increasing maternal mental health problems, such as clinical depression or anxiety, inadequate pharmacological treatment for chronic antenatal conditions and pregnancy undermonitoring.

Following this reasoning, there are other factors related to the pregnancy itself that might be affected by the consequences of COVID-19 and its management, including an elevated proportion of unplanned pregnancies [9], a high proportion of women who received at least one prescription during pregnancy (46–93%), with the most frequently prescribed drugs being those indicated for infections, pain conditions, heartburn and upper respiratory disorders such as antihistamines [10,11,12,13,14,15] and advanced maternal age. A study conducted in the US showed how the proportion of birth rates from women aged 35–39 years has increased from 45.9 per 1000 women in 2010 to 52.7 in 2019 [16,17]. This increase in maternal age might also lead to comorbidities, such as either pre-existing diabetes or gestational prediabetes, hypertension and hyperlipidemia, which require tighter antenatal care.

Focusing on guidelines and treatment recommendations during the COVID-19 pandemic, a number of medications have been recommended, such as corticosteroids (dexamethasone) and others approved, such as remdesivir, favipiravir, baricitinib and tocilizumab, together with others used off-label, such as lopinavir–ritonavir and high doses of hydroxychloroquine (HCQ). Worldwide clinical trials (CTs) have been carried out to build recommendations, such as the SOLIDARITY [18] and RECOVERY [19] CTs, promoted by the World Health Organization (WHO). Other recommendations include supportive pharmacological treatments with low molecular weight heparin [20,21,22]. Still, there is a gap of knowledge on the utilization patterns of these specific medications and this study could generate scientific knowledge to be considered on similar occasions that may occur in the future, for example, to stockpile the drugs for any potential next unknown emerged pathogens, to supply to pregnant women for the first priority. Therefore, we conducted a study with a retrospective cohort of pregnant women using real-world data from BIFAP (Pharmacoepidemiological Research Database for the Spanish Public Health System) to investigate the distribution of pregnant women with COVID-19 during the pandemic (2020–2022), together with the drug utilization patterns among pregnant women with and without (w/o) COVID-19 in order to see any difference. Finally, focusing on pregnant women with SARS-CoV-2 infection, we described the principal medications received during the COVID-19 infection, stratified by each wave and gestational age. 

## 2. Results

### 2.1. Distribution of Pregnant Women during the SARS-CoV-2 Pandemic

**Figure 1:**
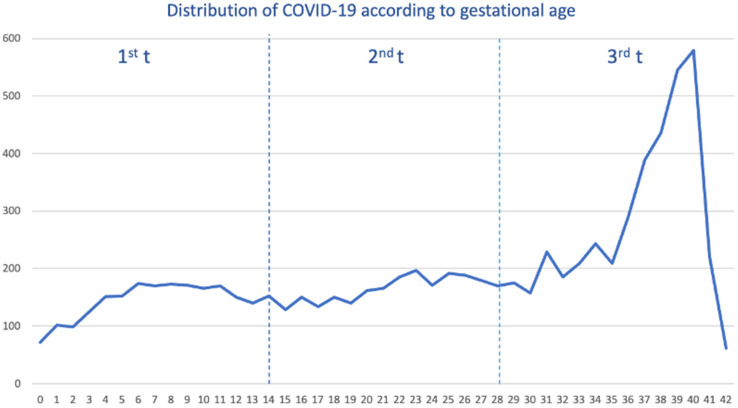
Distribution of pregnant women with COVID-19 by gestational week and trimester (t).

Out of a cohort of pregnant women, a total of 8413 tested positive for SARS-CoV-2, and we selected 24,975 pregnant women free of SARS-CoV-2. The median age was 32 years (interquartile range (IQR) 28–36 years). We saw the overall distribution of COVID-19 cases during the pandemic. As shown (Figure 1), the distribution of cases was constant from gestational week 6 to 36 weeks, with the highest peak occurring within 39 and 40 weeks. 

When we stratified by each pandemic wave (Supplemental Figure S1), the distribution of COVID-19 cases was heterogeneous. For example, within the 1st–2nd wave, the majority of cases occurred during the third trimester (3rd t), specifically in the last weeks of pregnancy, reaching their highest peak in week 40. In the third wave, COVID-19 cases were distributed in a smoothly staggered ascending pattern from the second (2nd t) to the third trimester, peaking in week 39, with very few cases within the first trimester (1st t). Contrary to that, from the fourth wave up to the sixth wave, the distribution of cases was completely homogeneous throughout the pregnancy, although the peak was also reached in weeks 39–40.

### 2.2. Drug Utilization Patterns during the SARS-CoV-2 Pandemic among Pregnant Women with and w/o COVID-19

**Figure 2:**
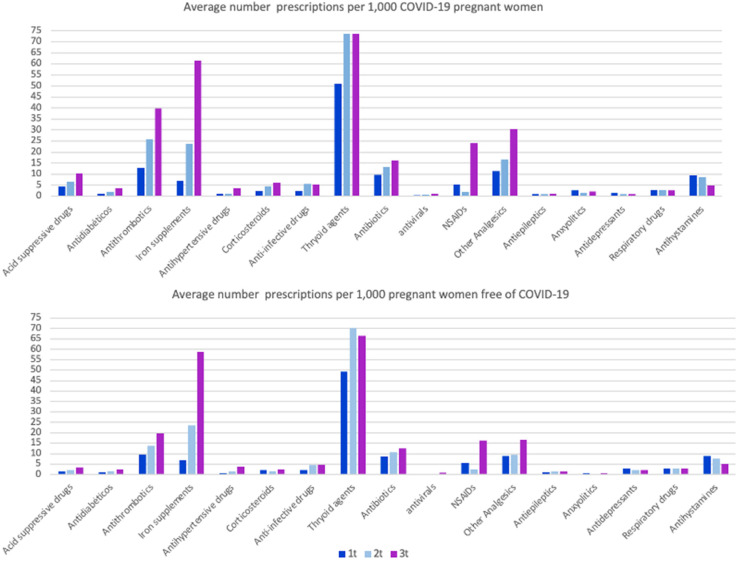
Average number of prescriptions per 1000 pregnant women and trimester (t), with and without COVID-19. Opioids, antifungal and constipation medications have been reviewed but not included in the graph, as the pattern of use is low (<2 prescriptions/1000 women per trimester) and similar in both groups.

Among pregnant women with and w/o COVID-19, we collected all prescriptions issued during the pregnancy, that is from the last menstrual period (LMP) date up to the end of pregnancy date, including chronic, symptomatic and/or etiologic treatments. Among women with COVID-19, a total of 6057 (72%) did receive at least one prescription, and 16,651 (66.6%) among women free of COVID-19. The average number of prescriptions was 8.1 prescriptions per woman among women with COVID-19 and 6.8 prescriptions among women without COVID-19, respectively. Secondly, we calculated the distribution of principal ATC groups (anatomical therapeutic chemical classification code assigned by the Collaborating Center for Drug Statistics Methodology of the WHO) expressed by average prescriptions per 1000 women among both groups, stratified by trimester of pregnancy (Figure 2).

The most prescribed therapeutic groups in both populations were thyroid agents, levothyroxine (66.1 prescriptions per 1000 pregnant women among those with COVID-19 group and 63.9 among those without COVID-19), followed by iron supplements (30.2 and 30.0), antithrombotic medications of the heparin group (heparin, dalteparin, enoxaparin, tinzaparin and bemiparin) (26.0 and 14.6), non-steroidal anti-inflammatory drugs (NSAIDs) (10.1 and 7.9), other analgesics (i.e., acetaminophen) (19.4 and 11.6), antibiotics (13.0 and 10.6) and antihistamines (7.8 and 7.2). As seen in Figure 2, the third trimester accumulated the majority of prescriptions, followed by the second trimester and, finally, the first trimester. Interestingly, for all pharmacological groups, pregnant women with COVID-19 received, on average, more prescriptions, with special attention to the average number of anxiolytics, which was almost fivefold higher among pregnant women with COVID-19 compared with those without (2.06 vs. 0.6), with the exception of antidepressants (1.07 and 2.45).

### 2.3. Drug Utilization Patterns within the Course of SARS-CoV-2 Infection

**Figure 3:**
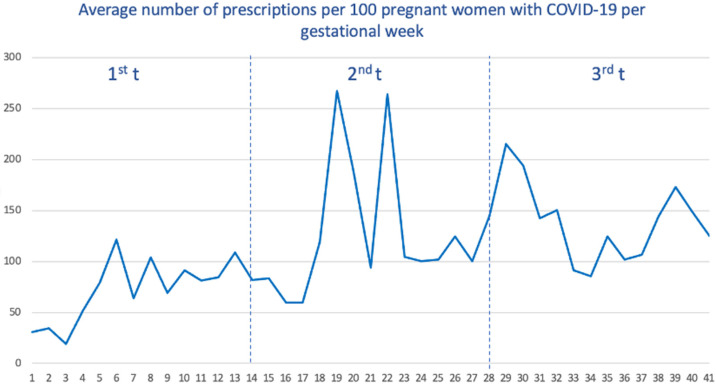
Average number of prescriptions per 100 pregnant women with COVID-19 by gestational age.

Among pregnant women with COVID-19, we calculated the average number of prescriptions received within the week before and during the week of SARS-CoV-2 infection. As seen (Figure 3), women who got the infection during the third trimester received, on average, a higher number of drugs (with a total average of 139 prescriptions per 100 women), followed by the second trimester (125) and the first trimester (77).

When we stratify by waves (Supplemental Figure S2), the first wave showed the lowest number of average prescriptions (138.4), and the vast majority of these prescriptions were accumulated when SARS-CoV-2 infection occurred in the third trimester. For the remaining waves, the distribution was more homogeneous, although the prescriptions received were also concentrated when SARS-CoV-2 infection occurred in the third trimester.

### 2.4. Patterns of COVID-19 Drug Utilization, Both Specific and Supportive

**Figure 4:**
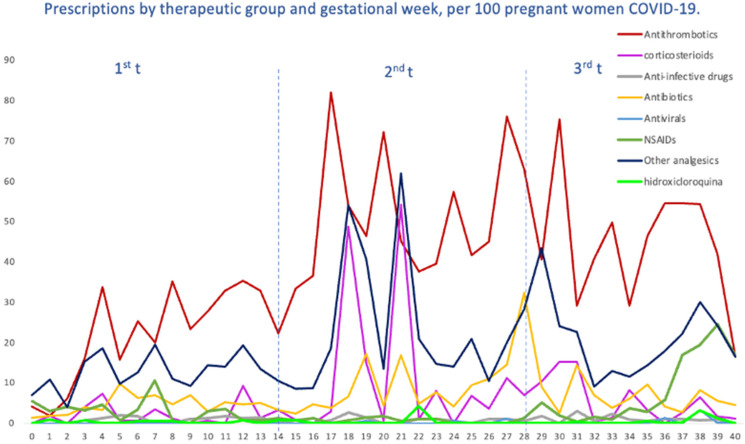
Prescriptions per 100 pregnant women with COVID-19, by therapeutic groups and by gestational week at the time of infection.

**Figure 5:**
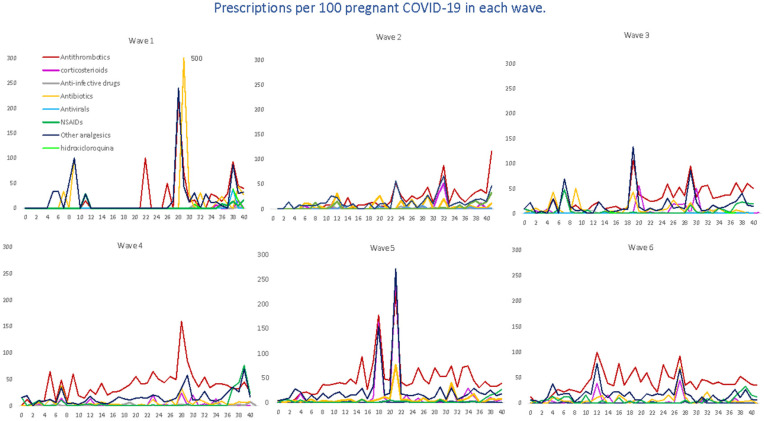
Average number of prescriptions per 100 pregnant women with COVID-19, by waves, therapeutic groups and the gestational week at the time of infection. Tocilizumab and opioids are not reported in the graph because they have only occasional prescriptions in some waves.

We evaluated the treatment patterns of COVID-19-specific medications, such as antivirals, corticosteroids, tocilizumab and HCQ, and medications used for supportive care, including antithrombotic agents, antibiotics, NSAIDs and other analgesics, in pregnant women with COVID-19, stratified by gestational age at SARS-CoV-2 infection. Overall, the highest average number of prescriptions was for antithrombotic medications of the heparin group, with 40 prescriptions per 100 pregnant women, followed by other analgesics (19.64), antibiotics (6.95), NSAIDs (6.29) and corticosteroids (5.80). The average number of prescriptions per 100 pregnant women for HCQ was 0.49, 0.31 for antivirals and 0.06 for tocilizumab (Figure 4). Likewise, in Figure 5, we can see the prescriptions segregated by each pandemic wave.

For antithrombotic medications, with enoxaparin representing 89% of all agents, the average number remained almost constant (from 36 to 45 prescriptions per 100 women) up to the fourth, fifth and sixth waves, where there was a dramatic increase in prescriptions, reaching more than 100 prescriptions. For corticosteroids, dexamethasone (22.67%) was the most prescribed systemic corticosteroid, followed by methylprednisolone (9.90%); we observed a heterogenous trend with ups and downs, with the highest average on the fifth wave. Antibiotics, represented by amoxicillin (23%), fosfomycin (20%), ceftriaxone (16.6%) and azithromycin (14.2%), were mostly used within the first wave (22 prescriptions per 100 women) and then gradually decreased, although there was a slight increase by the sixth wave (10.1); also, a similar trend was observed for other analgesics, 95% represented by acetaminophen (first wave 31 prescriptions per 100 women vs. 27.9 prescriptions per 100 women). Regarding the therapeutic group of NSAIDs, the most prescribed were Ibuprofen (66.08%) and Dexketoprofen (25.52%), reaching its maximum prescription peak in the fourth wave and its minimum in the fifth wave (76.74 prescriptions per 100 women vs. 26.74). However, regarding this last group, it should be noted that there were some peaks with occasional prescriptions, although non-prescription periods were predominant. Finally, the use of hydroxychloroquine was more frequent by the first and second waves, and then there was a decreasing trend as the waves advanced. Other specific drug treatments, such as tocilizumab or antivirals (only one prescription of remdesivir), were practically non-existent.

## 3. Discussion

The current study evaluated the distribution of COVID-19 during the pandemic among pregnant women, as well as treatment patterns and the principal medications prescribed to treat or palliate COVID-19 in this population. A total of 8413 pregnant women with COVID-19 were identified and 24,975 pregnant women free of COVID-19 were selected (1:3). According to the WHO, there have been five main variants of concern for SARS-CoV-2, starting with the first variants Alpha, Beta, Gamma, Delta and, finally, Omicron [23]. In our study, the distribution of COVID-19 cases during the pandemic has been gradually increasing from the first wave, with the lowest number of cases (348), to the fifth wave (Omicron), with the highest number of cases (1888), which expanded rapidly, followed by the second wave (1667). However, the distribution according to gestational age has been heterogenous, being concentrated within the last weeks of gestational age for the first and second wave, according to labor and delivery guidance for COVID-19 [24], and more homogeneously throughout the pregnancy for the remaining waves, where diagnostic and screening tests became available for health centers and self-diagnosis. 

In terms of drug utilization, we observed that a total of 72% of pregnant women with COVID-19 received a prescription, and 66.6% among women without COVID-19. Although there have been several factors that might affect the rates of drug prescriptions during the pandemic, such as public health policies, limited or inadequate healthcare prenatal visits, change in birth plan, limited care, quarantine and mobility restrictions [25,26,27,28], our results are in line with prior studies conducted before the COVID-19 outbreak, where they found a wide range in prevalence of receiving at least one prescription, from 27 to 93% [14]. The most relevant therapeutic groups prescribed in our study were analgesics (especially acetaminophen), antibiotics and thyroid hormones, in line with prior studies [29,30,31,32,33]. We found an inverse trend in drug prescriptions for psychotropic drugs; women with COVID-19 received more prescriptions for anxiolytics (benzodiazepines) and women without COVID-19 for antidepressants (selective serotonin reuptake inhibitors), leading to the possible risk of neonatal abstinence syndrome after delivery, which could have further complicated the scenario in the pandemic [34]. Several studies have reported elevated rates of adverse mental health problems during the COVID-19 outbreak, including anxiety, depression and post-traumatic stress symptoms, among others, [35,36,37,38] which might explain our findings. An international study conducted a cross-sectional survey of pregnant and postpartum women to report the impact of perinatal mental health during the pandemic [39]. The most reported worries included the pregnancy itself, the baby contracting COVID-19 and lack of support during labor. In addition, worries about children and missing medical appointments were associated with significantly higher odds of post-traumatic stress, anxiety/depression and loneliness. Recommendations that might improve mental health in pregnancy included providing accurate information, physical activity, social support, medical services and early diagnosis [40].

In terms of prescription rates of medication to treat or palliate COVID-19 in pregnant women with such diagnoses, in our study, antithrombotic medications of the heparin group were the most prescribed group, followed, in descending order, by other analgesics, antibiotics, NSAIDs, corticosteroids, HCQ, antivirals and tocilizumab. For example, the International Registry of Exposure to Coronavirus in Pregnancy [41] (IRCEP) reported as the most prescribed medications other analgesics, mainly acetaminophen, and antibiotics, predominantly azithromycin, and the COVID-19 International Pregnancy Drug Use Study, COVI-PREG [42], reported antibiotics as the most prescribed, mostly azithromycin, followed by corticosteroids, mostly represented by dexamethasone. Acetaminophen and dexamethasone also coincided as the most prescribed analgesic and systemic corticosteroid in our study; however, the most prescribed antibiotics were amoxicillin, fosfomycin, ceftriaxone and, in fourth place, azithromycin. The difference might be explained by different factors such as regional patterns of use, for example, the use of interferon in Russia due to patent or treatment availability and remdesivir in the US as the country of medical approval [41]. For antithrombotic medications, we observed a gradual increase compatible with the recommendations of the Spanish Society of Thrombosis and Hemostasis (SETH) on thromboprophylaxis in pregnant women launched in May 2020 [22]. There were very low prescription rates of hydroxychloroquine and antivirals and occasional prescriptions of tocilizumab. These prescriptions were accumulated within the first waves, and then there was a trend towards null rates coinciding with the publication of the results of the SOLIDARITY [18] clinical trial (October/2020), where the lack of efficacy of these drugs was reported. In contrast, corticosteroids (dexamethasone) started to be prescribed from the third trimester of the second wave, after the results of the RECOVERY [19] clinical trial were released.

The strengths of this study include the use of a large number of pregnancies, which is the result of applying a valid algorithm in a primary care database as described in the Section 4.3. Some limitations of the current study should be addressed. Although we used data from BIFAP, which is representative of the Spanish population with respect to age, sex and geographical region [43], only five regions provided data to perform COVID-19 studies. The data provided did not include the disease severity categorization; thus, sensitivity analyses according to each stage could not be performed. In addition, although we obtained data from primary care and also admissions to hospitals, due to low numbers of hospitalizations, intensive care unit (ICU) admissions and deceased, no stratified analyses according to these conditions were conducted in order to respond at a higher granularity. We could have missed some pregnancies and COVID-19 diagnoses due to several scenarios, including pregnancies that were followed by private clinics or with monitoring outside the primary care surgery and the under-recording of data in the electronic medical system due to an overload in the Spanish National Health System during years 2020 and 2022. No indication was recorded linked to each prescription, thus some of the medications reported within the week before or during the week of the COVID-19 diagnosis might have been prescribed for other purposes. In any case, this should be minimal, as we would not be able to observe any trend. An additional potential limitation to further explain results would be the lack of a previous period of SARS-CoV-2 for comparison.

BIFAP includes information based on prescription or dispensing, although this might not reflect actual drug intake, and therefore there is room for underestimation of the exposure, especially to over-the-counter medications. Finally, due to the study design being merely descriptive, we were not able to estimate the safety and effectiveness of specific drug treatments received to treat COVID-19 in this vulnerable population. Further studies are warranted in order to answer this question.

## 4. Material and Methods

### 4.1. Data Source

We conducted a retrospective cohort using data from BIFAP (Base de datos para la Investigación Farmacoepidemiológica en el Ámbito Público—Pharmacoepidemiological Research Database for Public Health System) which is owned by the Spanish Agency for Medicines and Medical Devices (AEMPS) and has the collaboration of the Autonomous Regions and the support of the main scientific societies involved. BIFAP is an anonymized electronic medical record database with a longitudinal, population-based focus for primary care practitioners (PCPs) and pediatricians. Currently, it includes information from demographic factors, consultation visits, referrals, hospital discharge diagnoses (and hospital/ICU admissions, but not for all regions systematically), laboratory test results, diagnostic procedures, diagnoses and prescriptions. At the time of the study, the database included information from nine participating Autonomous Regions (out of seventeen) in Spain. The distributions of age and sex are comparable with the Spanish population [44,45,46]. In terms of drug utilization, prescriptions issued by the PCPs are automatically recorded; prescriptions from specialists, as well as those used during hospitalizations, may not be fully captured. In addition, from 2011 onwards, e-prescription has progressively been implemented in primary care centers; therefore, dispensation is also available. Prescriptions are entered using the ATC classification. The quality of the data is ensured by maintaining high levels of scientific and technical quality of the projects carried out with the database. The BIFAP Scientific Committee is responsible for ensuring the quality of the information contained in the database. The information is then harmonized into the BIFAP data model; details on the BIFAP database have been described in detail previously [47].

### 4.2. Source Population

The source population included all women of childbearing age (15–49 years) with at least one year of registration with their PCPs, between January 2020 and December 2022, from five regions with SARS-CoV-2 data available upon the date of conducting the study. To be included in the study, women were required to be registered with their primary care physician at least one year before entering the study, to ensure previous information.

### 4.3. Identification of the Study Cohorts

We applied a valid algorithm [29] to identify all pregnancies occurring during the study period. This algorithm includes a first step which identifies indicators of pregnancy, such as: (i) indicators of conception, (ii) indicators of end of pregnancy and (iii) other codes compatible with a pregnancy, such as pregnancy tests, prenatal visits, pregnancy complications, etc. After the assignment of the validated gestational age, women identified as pregnant were classified according to pregnancy outcome into (i) term pregnancy, (ii) miscarriage, (iii) stillbirth or (iv) unspecific pregnancy, in which information did not allow to differentiate between the previous categories. Women whose gestational age could not be calculated (pregnancy with non-specific gestational age) were excluded. Once obtaining the cohort of pregnancies, we searched all pregnancies with a recorded diagnosis of COVID-19 during their gestational age, that is, from the last menstrual period up to the end of pregnancy (regardless of the outcome of pregnancy). A patient was classified as a confirmed case if they met one of the following criteria: a confirmed case of SARS-CoV-2 infection from the active surveillance system implemented during the COVID-19 pandemic and from hospital data or from an ICU [44]. For each pregnant woman with a confirmed diagnosis of COVID-19 (N = 8413), we matched 3 pregnant women without COVID-19 with the same age at the date of SARS-CoV-2 diagnosis (+/2 years), same gestational age (+/− 2 weeks) and length of pregnancy and outcome (N = 24,975).

### 4.4. Drug Exposure

In order to give an answer to our study aims, we followed two different strategies to collect the drug utilization information. The prescriptions were based either on recordings in the database or pharmacy dispensing, when available. We looked at all drug prescriptions issued in their medical records, including primary and hospital care, using two different time frames: (i) all prescriptions received throughout pregnancy, that is, from the LMP date up to the end of pregnancy date and (ii) restricting to women with COVID-19, we searched for all drug prescriptions issued within the week prior to the SARS-CoV-2 diagnosis and within the week following the diagnosis. This time was used to ensure inclusion of all prescriptions issued when symptoms were triggered before the diagnosis of SARS-CoV-2 and, therefore, to avoid an underestimation of use. Medication use was considered if at least one prescription was recorded within each time frame given.

### 4.5. Statistical Analysis

First, we conducted a descriptive analysis of the number of pregnant women with a diagnosis of COVID-19 according to each SARS-CoV-2 wave. To establish each wave, we followed the classification used by the National Epidemiological Surveillance Network (RENAVE) through the SiViES (System for Surveillance in Spain) platform, managed by the National Epidemiology Center [48] (CNE). Therefore, the first wave was established from 02/2020 to 22/05/2020, the second from 06/07/2020 to 18/11/2020, the third from the beginning of December/2020 to 16/02/2021, the fourth from 02/04/2021 to 17/05/2021, the fifth from 01/07/2021 to 29/09/2021 and the sixth from 13/11/2021 to 28/03/2022. Second, we described the pharmacologic treatments prescribed during the pandemic among pregnant women with and without (w/o) COVID-19, stratified by trimester, considering the first trimester up to week 13, the second trimester from 14 to 28 and the third trimester from week 28 onwards. We reported the medication use as the average of prescriptions per 1000 pregnant women, using as the numerator the number of prescriptions divided by the total number of included pregnancies as the denominator. Lastly, we reported the drug utilization patterns restricted to pregnant women with SARS-CoV-2 infection. We assessed the overall drug use within the week prior to and after the diagnosis, stratified by each pandemic wave as well as COVID-19-specific medications, defined as pharmacological agents to potentially have effects against COVID-19 or supportive care, such as tocilizumab, hydroxychloroquine, antithrombotic medications of the heparin group, antivirals, antibiotics, NSAIDs, analgesics and corticosteroids. We also reported the medication use as the average of prescriptions per 100 pregnant women. We examined patterns of medication use by gestational age (in weeks) and the COVID-19 wave. Due to low numbers of hospitalizations (503/8413; 6%), ICU (4/8413), and deceased (2/8413), no stratified analyses according to these conditions were conducted.

## 5. Conclusions

In summary, the distribution of COVID-19 among pregnant women followed a gradual increase, reaching its peak in the fifth and second wave, in line with the propagation of each SARS-CoV-2 variant. Pregnant women, regardless of SARS-CoV-2 infection, appear to have received similar prescription rates compared to the studies of pre-pandemic period drug utilization consulted.

The most common drugs used in pregnant women with and without COVID-19, during the pandemic, have been thyroid hormones (levothyroxine), analgesics (acetaminophen mostly), antithrombotics of the heparin group (enoxaparin mainly), antibiotics (amoxicillin and fosfomycin the most commonly used) and corticosteroids (dexamethasone for systemic use is the most commonly used), as well as iron supplements. Among pregnancies with COVID-19, there was a small rate of prescribing specific medications, in line with scientific evidence and clinical recommendations.

According to the European Medicines Agency (EMA), it is good practice to always try to collect information on medicine exposure during pregnancy [49]. Studies on the use of drugs in pregnancy are important to understand the safety and risk of using specific medicines during pregnancy. The EMA has issued guidelines [49] on the exposure to medicinal products during pregnancy, which include recommendations for monitoring exposure during the entire period and providing data on exposure to medicinal products. This research on the use of drugs during pregnancy in the pandemic—under exceptional conditions against an unknown pathogen—will contribute to knowing the specific drugs that have been used for pregnant women with COVID-19, providing information to both healthcare professionals and pregnant women, to assess the potential risk–benefit balance and to make the best decision on pharmacological treatment.

## Data Availability

Individual data from the BIFAP-database cannot be made publicly available. For the dataset, the BIFAP-database can be contacted at the following email-address: bifap@aemps.es.

## Supplementary Materials

The following supporting information can be downloaded at: https://www.mdpi.com/article/10.3390/ph17020207/s1, Figure S1: Distribution of pregnant women with COVID-19 by gestational week stratified by pandemic waves; Figure S2: Average number of prescriptions per 100 pregnant women with COVID-19 by gestational age, stratified by pandemic waves.
