# Supplementary figures and images for: COVID-19 Distribution in Pregnancy, Drug Use Patterns and COVID-19 Medication during the Pandemic in Spain: Data from Real-World Electronic Health Records
Source: Pharmaceuticals (Basel). 2024 Feb 6;17(2):207. doi: 10.3390/ph17020207 (PMC10892820; doi:10.3390/ph17020207)

Wave 1

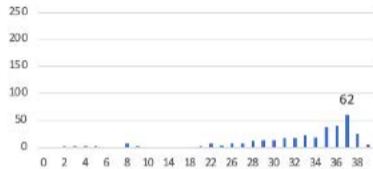

Wave 2

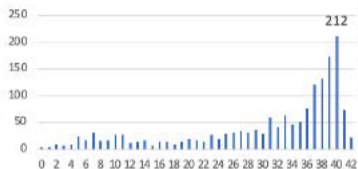

Wave 3

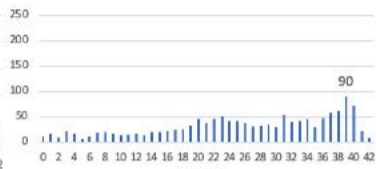

Wave 4

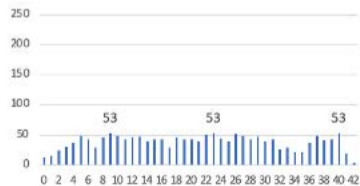

Wave 5

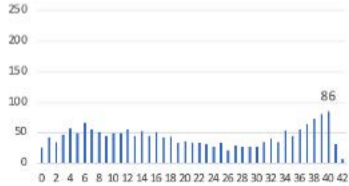

Wave 6

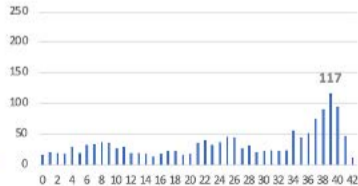

Supplement: Supplementary file 1 [file pharmaceuticals-17-00207-s001.zip › Figure S1.pdf]

### First Wave

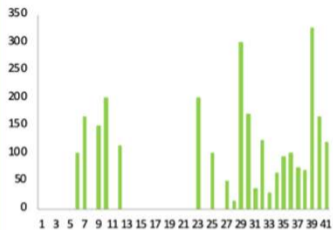

### Second Wave

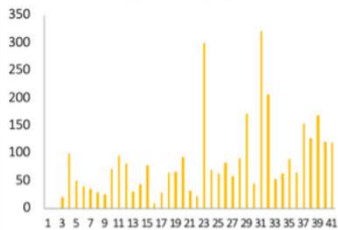

### Third Wave

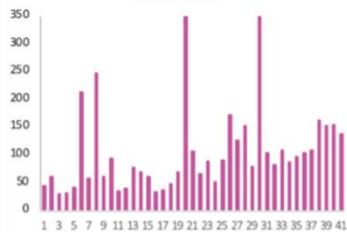

### Forth Wave

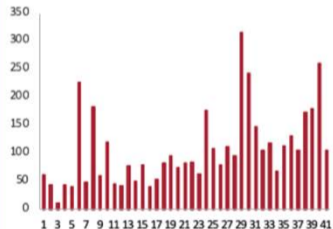

### Fifth Wave

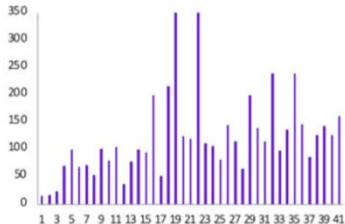

### Sixth Wave

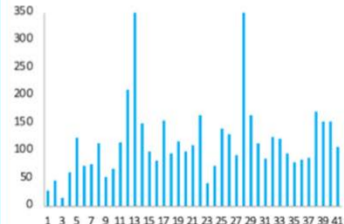

Supplement: Supplementary file 1 [file pharmaceuticals-17-00207-s001.zip › Figure S2.pdf]
